# Supplementary material for: Effectiveness of Gold Nanorods of Different Sizes in Photothermal Therapy to Eliminate Melanoma and Glioblastoma Cells
Source: Int J Mol Sci. 2023 Aug 27;24(17):13306. doi: 10.3390/ijms241713306 (PMC10488215; doi:10.3390/ijms241713306)
Supplement: Supplementary file 1 [file ijms-24-13306-s001.zip › Supplementary Information.pdf]

Supplementary Information

Effectiveness of gold nanorods of different sizes in photothermal therapy to eliminate melanoma and glioblastoma cells.

Javier Domingo-Diez[1], Lilia Souiade[1], Vanesa Manzaneda-González[2], Marta Sanchez[1,3], Diego Megias[4], Andrés Guerrero-Martínez[5], Carmen Ramírez[1,3,5,6], Javier Serrano-Olmedo[1,7,8], Milagros Ramos-Gómez[1,7,8,*]

[1]Center for Biomedical Technology (CTB), Universidad Politécnica de Madrid (UPM), 28040, Madrid, Spain

[2]Departamento de Química Física, Universidad Complutense de Madrid, Avenida Complutense s/n, 28040 Madrid, Spain

[3]Grupo de Sistemas Complejos, Universidad Politécnica de Madrid, 28040 Madrid, Spain

[4]Advanced Optical Microscopy Unit, UCCTs, Instituto de salud Carlos III (ISCIII), E28220 Majadahonda, Madrid

[5]Departamento Biotecnología-B.V. ETSIAAB, Universidad Politécnica de Madrid, 28223 Pozuelo de Alarcón, Spain

[6]Departamento de Oncología. Instituto de Investigación Sanitaria San Carlos, (IdISSC), 28040 Madrid, Spain

[7]Centro de Investigación Biomédica en Red para Bioingeniería, Biomateriales y Nanomedicina, Instituto de Salud Carlos III, Spain

[8]Departamento de Tecnología Fotónica y Bioingeniería. ETSI Telecomunicaciones, Universidad Politécnica de Madrid, 28040 Madrid, Spain

* Corresponding author at: Milagros Ramos-Gómez, Experimental Neurology Unit. Center for Biomedical Technology. Universidad Politécnica de Madrid. Campus de Montegancedo S/N, Pozuelo de Alarcón, 28223, Madrid, Spain.

E-mail address: milagros.ramos@ctb.upm.es

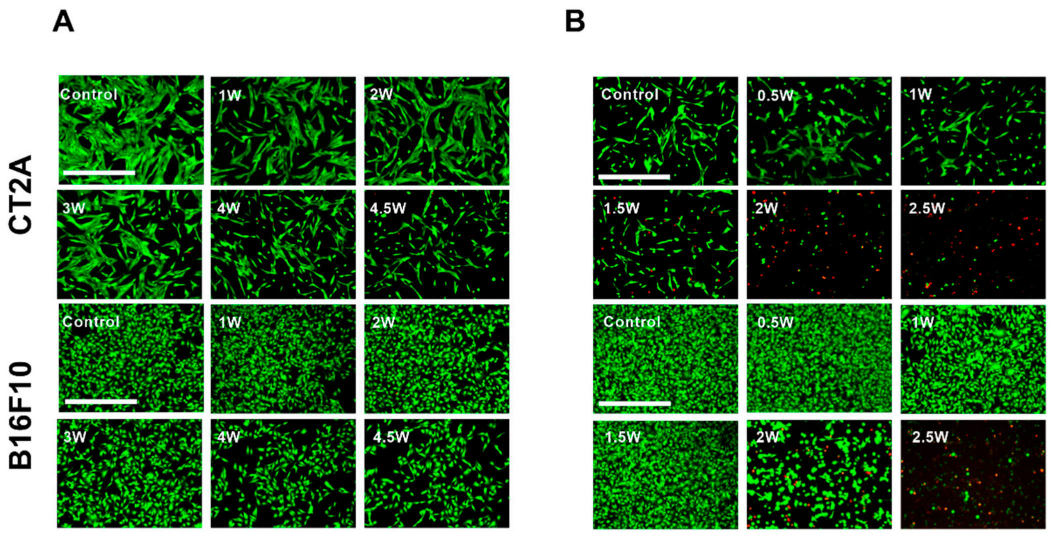

Supplementary Figure S1. Viability of CT2A and B16F10 cells evaluated by the calcein/PI assay 24 h after irradiation with an 808 nm laser for 10 min at the indicated powers. Cells were irradiated at RT (A) and at 37 °C (B). Fluorescence microscopy images show living cells in green stained with calcein and dead cells in red stained with PI. Scale bars: 400 µm

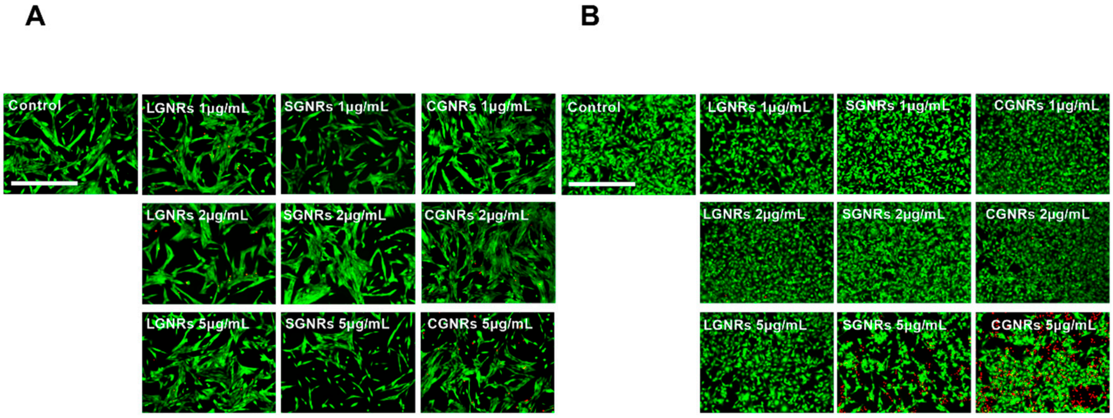

Supplementary Figure S2. CT2A (A) and B16F10 (B) cell viability determined by the calcein/PI assay after incubation with increasing concentrations of LGNRs, SGNRs and CGNRs for 24 h. Fluorescence microscopy images show living cells in green stained with calcein and dead cells in red stained with PI. Scale bars: 400 µm.

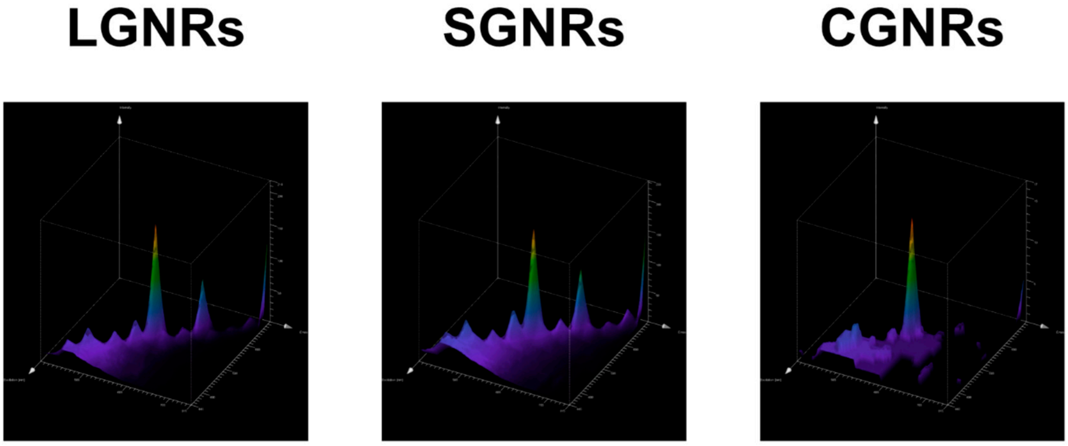

Supplementary Figure S3. 3D Autofluorescence emission and excitation profiles of the different GNRs by confocal microscope. All types of GNRs were detected by reflection under excitation with a laser line of 488 nm and by collecting the emission in the 480–500 nm range.

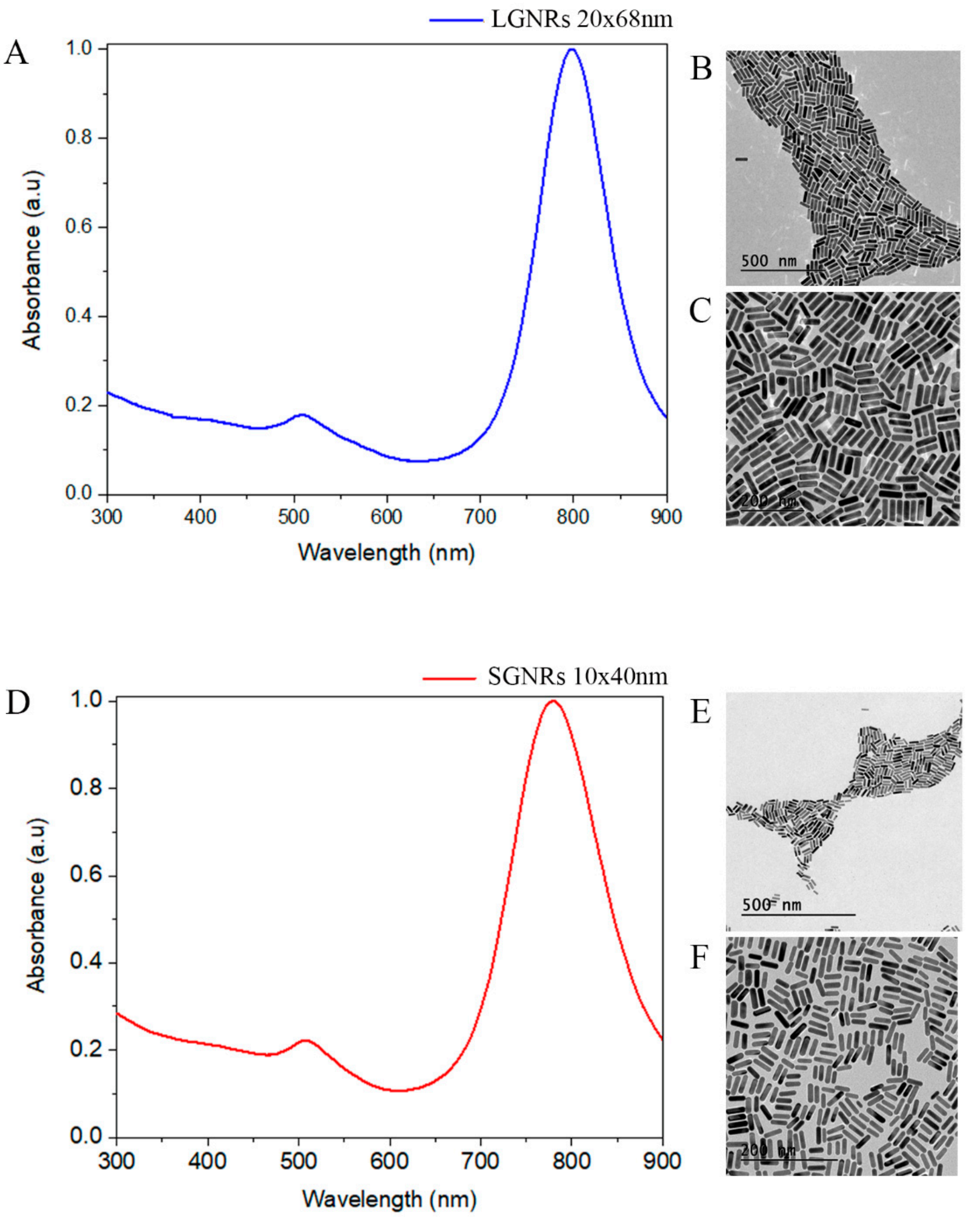

Supplementary Figure S4. Peak of absorbance at 808 nm of LGNRs (A) and SGNRs (D). Images TEM of LGNRs (B-C) and SGNRs (E-F) with different magnifications.
